# Psychometric models of emerging adulthood: an evaluation in a Chilean university sample

**DOI:** 10.3389/fpsyg.2025.1483934

**Published:** 2025-04-25

**Authors:** Paula Boero, Italo Trizano-Hermosilla, Eugenia V. Vinet

**Affiliations:** Departamento de Psicología, Universidad de La Frontera, Temuco, Chile

**Keywords:** emerging adulthood, university students, validity, reliability, ESEM

## Abstract

This study analyzed three models of the Inventory of the Dimensions of Emerging Adulthood (IDEA): the original by Reifman et al., the version based on Arnett’s proposals, and the abbreviated version by Crocetti et al. The sample included 1935 students from four Chilean universities (56% women), with an average age of 21.3 years (SD = 2.04). The 31 items of the instrument were descriptively analyzed, followed by analyses to determine the best-fitting factorial model. Confirmatory Factor Analyses and Exploratory Structural Equation Modeling were utilized. Finally, reliability estimates were obtained. The results showed that Crocetti et al.’s model offered the best fit, consistent with theoretical postulations, and acceptable reliability levels, proving to be the best of the evaluated models. This version confirmed five correlated latent dimensions, providing an integrated interpretation of the Emerging Adulthood construct for use in the Chilean population.

## 1 Introduction

Emerging adulthood (EA), initially proposed by Arnett (2000), is a construct that has been widely used to characterize the period of life between 18 and 29 years of age, coinciding with the years in which young people pursue different tertiary education alternatives, and at the same time, seek to satisfy various personal needs and motivations. Initially, Arnett (2000) described it as a demographically distinctive period where “nothing is normative” (p. 471) with diverse life trajectories.

As with many psychological constructs, an instrument was developed to describe and measure different dimensions of EA. The first proposal was developed by Reifman et al. (2007) and included the five distinctive features of EA originally conceptualized by Arnett (2000, 2004). Namely, EA is seen as the age of: identity explorations; instability/negativity; self-focused; feeling “in between” adolescence and adulthood; and experimentation and possibilities. This proposal also included a sixth, non-theoretical, dimension, others-focus, as a complementary continuum from the dimension of self-focused.

Psychometrically, Reifman et al. (2016) expressed concern that items assigned to one subscale of the IDEA tended to correlate with items from other subscales, resulting in a high degree of correlation between subscales. Thus, the development of the IDEA inventory, and its psychometric characteristics, has been somewhat controversial, as different items have been assigned to subscales other than those in which they had the highest absolute factor loadings. In this regard, Reifman et al. (2016) suggest that theoretical-conceptual criteria have prevailed over psychometric ones, in defining the composition of the IDEA subscales. According to Reifman et al. (2016) observations, it is understandable that the translations and adaptations of the inventory, developed in different countries and different cultural contexts, have not been able to fully reproduce the original structure of the IDEA.

As reported by Yerofeyeva et al. (2024) the IDEA has been adapted in several countries, but its factor structure varies from the original version, making it difficult to compare emerging adulthood characteristics across cultures. The authors noted that in some countries, such as the Netherlands, ethnic-specific versions have been developed, while in others, such as Greece, Spain, Mexico, Chile, and Russia have undergone independent psychometric analyses with mixed results. In Chile, Pérez et al. (2008) explored the factorial structure of the IDEA, finding a four-factor solution, two of which were consistent with the original solution by Reifman et al. (2007), a third one combined aspects of identity exploration and feeling “in between,” and a fourth factor in which items related to self-focused and others-focus appeared together. Furthermore, measurement invariance studies have shown inconsistencies, finding different models in culturally similar countries, such as Spain and Mexico (Fierro and Moreno, 2007), and identical models in culturally different countries, such as Italy and Japan (Crocetti et al., 2015). Additionally, Yerofeyeva et al. (2024) indicate that the five original factors of the model have not always been confirmed, being reduced to three or four dimensions and with different numbers of items. This situation occurs in versions from Greece (Leontopoulou et al., 2016), Switzerland (Baggio et al., 2015), Turkey (Atak and Çok, 2008), Chile (Pérez et al., 2008), Spain (Sánchez-Queija et al., 2020), China (Kuang et al., 2023), and Malaysia (Wider et al., 2016). An exception is the study by Yerofeyeva et al. (2024) in Russia, China, and Armenia, where five dimensions with 21 items were found.

The difficulty of having a single instrument to attempt comparability of EA studies has been overcome in a pragmatic and conceptually respectful way by Crocetti et al. (2015). These authors start from the items of the original IDEA and propose a shorter instrument called IDEA-Short Version (IDEA-SV). In it, the other-focus dimension was eliminated due to psychometric inconsistencies and because that dimension is not part of Arnett’s (2000, 2004) original theory. In addition, considering the psychometric information from the original IDEA (Reifman et al., 2007), they reduced the items of each dimension to overcome psychometric deficiencies such as unsatisfactory fit indices in Confirmatory Factor Analysis (CFA), cross-loadings between dimensions and items, and high correlations between residual errors.

The IDEA-SV consisted of 15 items, three for each dimension. The retained items were chosen for their conceptual coherence with the measured dimensions; moreover, psychometrically they correspond to the three highest factor loadings found in each dimension of the original IDEA. The adequacy of the IDEA-SV was verified in a sample of 2,472 Italian and Japanese emerging adults, male and female, aged 18–30 years, which included university students and workers. Using CFA, the IDEA-SV was tested to fit well across samples by country; furthermore, through three-level analyses of measurement invariance (configurational, metric, and scalar), comparability of latent variables between national groups of students and workers was ensured (Crocetti et al., 2015).

The psychometric analysis techniques of the IDEA have been changing during its development, from the first version in 2007 to the current ones (e.g., Crocetti et al., 2015; Yerofeyeva et al., 2024). Reifman et al. (2007), used as a starting point the principal components analysis (PCA) and varimax rotation, which was corroborated by a CFA that confirmed decisions based mainly on theoretical assumptions. Subsequent studies employed their analysis strategies using variations of PCA and CFA until the study by Crocetti et al. (2015) who applied CFA supplemented with invariance analysis to ensure comparability between samples.

The current study proposes to advance psychometric analysis tools by introducing Exploratory Structural Equation Modeling (ESEM, Asparouhov and Muthén, 2009). Traditionally, CFA assumes that the loadings of items not theoretically assigned to a factor should be set to zero, an assumption that may be unrealistic in psychometric practice. The ESEM addresses this problem by allowing free estimation of such loadings, which may be more representative of real measurement conditions in psychology and, at the same time, it provides a better model of fit while maintaining the overall structural logic in line with the theoretical approaches of the instrument (Marsh et al., 2014, 2020). In addition, the ESEM has proven to be superior to the CFA approach for investigating item factor analysis solutions, where cross-loadings are the rule rather than the exception (Hong et al., 2024; Gomes and Gjikuria, 2017). A significant problem with the CFA approach is that it tends to overestimate the correlations between factors since all loadings of non-target items are carried over to the factor correlations, producing an incorrect estimate of the factor correlations (Gomes and Gjikuria, 2017; Gomes et al., 2017).

Stemming from the background reviewed so far, this paper sought to evaluate three IDEA models for use in the Chilean university context: (1) the original model by Reifman et al. (2007); (2) the theoretical proposal from Arnett (2000, 2004); and (3) the IDEA-SV proposed by Crocetti et al. (2015), using, in each case, CFA and ESEM.

Consequently, the first objective of this study was to evaluate and contrast three IDEA models: the original one by Reifman et al. (2007), the theoretical proposal by Arnett’s (2000, 2004), and a reduced model proposed by Crocetti et al. (2015) using CFA and ESEM in a large sample of Chilean university students; and its second objective was to estimate the reliability levels of the model that obtains the best fit for the data.

Therefore, the hypotheses are:

H1: Crocetti’s model (IDEA-SV, 15 items, and 5 dimensions) adequately explains the relationships between the items in Chilean university students.

H2: Estimates obtained using the ESEM psychometric model produce better fits than the CFA.

H3: The correlations between the IDEA factors obtained through the ESEM are attenuated compared to those estimated through CFA.

H4: The reliability levels obtained in the IDEA-SV dimensions are adequate for research purposes.

## 2 Materials and methods

### 2.1 Participants

A purposive sampling method was used to select 1,935 undergraduate university students that met the inclusion criteria of being emerging adults (aged 18–29 years) and Chilean nationals. The participants had a mean age of 21.30 years (SD = 2.04), with 56% being female. They were drawn from public universities in Chile. Socioeconomic status (SES) was classified through self-report using the ESOMAR criteria (Adimark, 2000), resulting in the following distribution: low (28.5%), middle (30.1%), and high (41.4%). The distribution by field of study was as follows: engineering (31.6%), health (29.9%), education and social sciences (25.6%), and other areas (12.9%).

### 2.2 Procedure

The students were contacted through their professors or program directors, who authorized the administration of a survey that included, among other instruments, the original IDEA, from which the items for the IDEA-SV were later extracted. Before administration, the students were informed about the research purpose and the voluntary nature of their participation. Those who agreed to participate signed an informed consent form approved by the Scientific Ethical Committee of the University of La Frontera. The survey was administered in groups of approximately 30 participants, using either paper or online versions depending on the availability of facilities, and was supervised by researchers and their assistants. The activity took approximately one hour.

### 2.3 Measures

The survey comprised a socio-demographic questionnaire that recorded self-reported variables such as age, sex, major, and SES, along with a set of measures characterizing the students in various sociocultural and mental health variables relevant to EA.

The IDEA version used in this study is Reifman’s (2007) original, ensuring strict adherence to the original content of the items. The previous work by Pérez et al. (2008), conducted in Chile, was a reference in the final wording of the items used in this study. This Chilean version consists of the 31 original items grouped into the six dimensions proposed by Reifman et al. (2007). Subsequently, the items selected by Crocetti et al. (2015) for the IDEA-SV were identified. Both, the 28-item IDEA and the IDEA-SV, measure the core characteristics of EA as coined by Arnett (2000, 2004) and are shortened versions of the original Reifman et al. (2007) instrument. Items are rated on a four-point scale ranging from 1 (strongly disagree) to 4 (strongly agree). Scores reflect the average of the items in each dimension, with higher scores indicating greater identification with the measured characteristic.

### 2.4 Data analysis

In the initial phase, a detailed descriptive analysis was conducted for the 31 items of the instrument. These evaluations provide a basic understanding of the data characteristics.

Subsequently, the best-fitting factorial model for the IDEA data was determined. CFA and ESEM were used to assess the internal structure (Alamer, 2022), contrasting the three proposed models for explaining the relationships among the IDEA items: (1) the original six-factor, 31-item model by Reifman et al. (2007); (2) Arnett’s five-factor, 28-item theoretical model; and (3) Crocetti et al.’s (2015) reduced five-dimensional, 15-item model.

Given the ordinal nature of the items and the results of the descriptive analyses, polychoric correlation matrices and the WLSMV estimation method were employed. This approach is particularly suitable for data with few response categories and high skewness (Verhulst and Neale, 2021). The fit indices used to evaluate model quality included the Comparative Fit Index (CFI), Tucker-Lewis Index (TLI), Root Mean Square Error of Approximation (RMSEA) with its 90% confidence interval, and the Standardized Root Mean Square Residual (SRMR), the latter being particularly recommended (Shi and Maydeu-Olivares, 2020). These analyses were performed using Mplus 8.1 software.

Finally, considering the current recommendations (see Flora, 2020), once the model with the best fit indices was identified, reliability estimates for the IDEA scores were obtained. Given the limitations of Cronbach’s alpha (Fu et al., 2022; Trizano-Hermosilla and Alvarado, 2016), McDonald’s (1999) omega coefficient was estimated. Considering the standards proposed by the American Educational Research Association [AERS] (2014), acceptable reliability values were considered, particularly in the context of research and at an initial stage, with values above 0.60. Reliability analyses were conducted using FACTOR 12.04 software (Ferrando and Lorenzo-Seva, 2017).

## 3 Results

### 3.1 Descriptive analysis of items

Table 1 presents the descriptive statistics of the 31 items of the IDEA, the original version of Reifman et al. (2007), which also includes the 28-item version of Arnett’s (2000) theoretical model, and the 15 items of the short version of Crocetti et al. (2015). The table shows that item 7 presents the highest mean (M = 3.64; SD = 0.53) and item 6 the lowest (M = 2.02; SD = 0.81). The median and mode for most of the items are consistently 3 and 4, indicating a general tendency toward responses in the medium-to-high range. The skewness of the items also varies: item 28 shows the highest negative skewness (−1.24) and item 6 the highest positive skewness (0.45), indicating differences in the symmetry of the distribution of responses, which go in the same relationship as the items with the highest and lowest means.

**TABLE 1:** Descriptive statistics of the 31 IDEA items.

Items	Mean	Median	Mode	SD	Skewness	Kurtosis
1	3.43	4	4	0.66	−0.92	0.55
2	3.30	3	3	0.66	−0.66	0.36
3	2.69	3	3	0.88	−0.19	−0.68
4	3.19	3	3	0.74	−0.71	0.31
5	3.03	3	3	0.81	−0.57	−0.13
6	2.02	2	2	0.81	0.45	−0.32
7	3.64	4	4	0.53	−1.20	1.05
8	2.95	3	3	0.84	−0.40	−0.50
9	2.37	2	2	0.86	0.09	−0.65
10	3.31	3	3	0.67	−0.67	0.32
11	3.14	3	3	0.79	−0.63	−0.13
12	3.07	3	3	0.87	−0.63	−0.35
13	2.78	3	3	0.83	−0.21	−0.56
14	2.21	2	2	0.91	0.36	−0.66
15	3.07	3	3	0.77	−0.54	−0.03
16	3.45	4	4	0.63	−0.89	0.66
17	2.86	3	3	0.80	−0.32	−0.35
18	2.68	3	3	0.76	−0.23	−0.24
19	3.18	3	3	0.70	−0.56	0.12
20	2.91	3	3	0.78	−0.22	−0.56
21	3.22	3	3	0.69	−0.55	0.11
22	3.22	3	3	0.69	−0.57	0.14
23	2.51	2	2	0.86	0.05	−0.64
24	3.19	3	3	0.71	−0.69	0.55
25	3.32	3	3	0.70	−0.81	0.37
26	3.10	3	3	0.70	−0.53	0.39
27	2.88	3	3	0.94	−0.48	−0.65
28	3.42	4	4	0.73	−1.24	1.37
29	2.98	3	3	0.89	−0.55	−0.45
30	3.24	3	3	0.70	−0.73	0.62
31	2.74	3	3	0.83	−0.34	−0.36

SD, standard deviation; standard error of skewness = 0.06; standard error of kurtosis = 0.11.

### 3.2 Psychometric evaluation of the three IDEA’s models

Table 2 shows the fit indices for the different models evaluated. In general, it is observed that the original model of Reifman et al. (2007) and the Arnett model present poor fit in the CFA approximation; however, their fit improves when evaluating the same model in its ESEM approximation. Crocetti et al.’s (2015) model shows an almost acceptable fit in its CFA version and the best good of fit from among all tested models in its ESEM approximation. Therefore, this model is the one that will be used to estimate the reliability of the scores.

**TABLE 2:** Fit indexes for three IDEA models.

Analysis	Model	χ^2^	gl	CFI	TLI	RMSEA	SRMR
CFA	1	6,421.18	419	0.758	0.732	0.086 (0.084–0.088)	0.084
	2	5,391.03	340	0.782	0.758	0.088 (0.086–0.090)	0.084
	3	871.59	80	0.912	0.884	0.072 (0.067–0.076)	0.055
ESEM	1	1,815.49	294	0.939	0.903	0.052 (0.049–0.054)	0.029
	2	1,828.48	248	0.932	0.896	0.057 (0.067–0.076)	0.032
	3	165.32	40	0.986	0.963	0.040 (0.034–0.047)	0.017

CFA, confirmatory factor analysis; ESEM, exploratory structural equation modeling. 1 = Reifman et al., 2007 Model; 2 = Arnett, 2000 Model; 3 = Crocetti et al., 2015 Model; χ^2^ = WLSMV-chi-square. CFI, Comparative FIt Index; TLI, Tucker Lewis Index; RMSEA, Root Mean Square Error of Approximation; SRMR, Standardized Root Mean Squared Residual.

### 3.3 Factorial loadings from the ESEM

Table 3 presents the estimates of the factorial loadings of the ESEM for the short version of 15 items, showing that each item has loadings greater than 0.40 in the factors that theoretically correspond (marked in bold). At the same time, small loadings, all less than 0.30, are observed in those factors where loading does not theoretically correspond. These values are consistent with what was expected, reaffirming the structure of the scale.

**TABLE 3:** Factor loadings of the ESEM IDEA-SV.

Items*	IDEN	NEG	SF	INB	EXP
24	**0.424**	−0.026	0.202	0.114	0.156
8	−0.007	**0.823**	−0.098	0.028	−0.023
7	−0.022	0.113	**0.416**	0.096	0.023
29	0.000	−0.008	−0.015	**0.788**	−0.034
1	−0.051	−0.073	0.205	−0.039	**0.546**
27	**0.899**	−0.008	−0.054	−0.078	−0.051
11	−0.021	**0.839**	0.026	−0.001	0.106
15	0.022	−0.061	**0.599**	−0.024	0.045
30	0.001	−0.004	0.291	**0.502**	0.009
2	0.017	0.045	−0.083	−0.015	**0.897**
28	**0.532**	0.039	0.193	0.102	0.059
20	0.029	**0.720**	0.077	−0.027	−0.064
19	0.020	0.048	**0.728**	−0.133	−0.035
31	0.066	0.158	−0.133	**0.446**	0.016
21	0.121	−0.013	0.153	0.101	**0.486**

*Item numbering according to Reifman et al., 2007; ordering according to Crocetti et al., 2015. In each column, the highlighted values (in bold) are those that represent the construct measured by that column. IDEN, identity exploration; NEG, instability/negativity; SF, self-focused; INB, feeling-in-between; EXP, experimentation/possibilities.

### 3.4 Descriptive statistics, reliabilities, and correlations between factors

The main features obtained in this section are summarized in Table 4. It first presents the means and SDs obtained in the IDEA-SV showing that the scores of the dimensions vary between 3.32 (SD = 0.51), in experimentation/possibilities, and 2.99 (SD = 0.58), for feeling-in-between. Next, the reliabilities obtained by Omega are presented. Variability in the precision of the scores from the dimensions is observed. On the one hand, high levels of reliability are observed for instability/negativity (0.84) and moderate levels for the other dimensions, fluctuating between 0.61 and 0.69.

**TABLE 4:** Descriptive statistics, reliabilities by dimension, and correlations between dimensions of the IDEA-SV ESEM (correlations from the CFA model are presented in parentheses).

IDEA DIM	M (SD)	Omega	1	2	3	4
1 IDEN	3.16 (0.61)	0.67	–			
2 NEG	3.00 (0.67)	0.84	0.09** (0.10**)	–		
3 SF	3.30 (0.48)	0.61	0.22*** (0.40***)	0.03 (0.05)	–	
4 INB	2.99 (0.58)	0.61	0.33*** (0.49***)	0.23*** (0.31***)	−0.01 (0.04)	–
5 EXP	3.32 (0.51)	0.69	0.44*** (0.56***)	−0.12** (−0.10**)	0.37*** (0.40***)	0.23*** (0.30***)

IDEA DIM = IDEA-SV; 1 = identity exploration, 2 = instability/negativity, 3 = self-focused, 4 = feeling-in-between, 5 = experimentation/possibilities. M, mean; SD, standard deviation.

**p < 0.05,

***p < 0.001.

Finally, the correlations between the dimensions of the IDEA-SV are presented. The results revealed both positive and negative relationships among the dimensions. Among the former, a moderately high correlation was found between identity exploration and experimentation/possibilities (0.44); experimentation/possibilities and self-focus (0.37); and identity exploration and feeling in-between (0.33). Moderate correlations were also observed between feeling in-between and instability/negativity (0.23), feeling in-between and experimentation/possibilities (0.23), and self-focus and identity exploration (0.22). A weak correlation was found between instability/negativity and identity exploration (0.09), and a weak negative correlation between instability/negativity and experimentation/possibilities (−0.12). Overall, the correlations estimated with the CFA model were higher than those obtained through ESEM (see Table 4).

## 4 Discussion

This study aimed to evaluate and contrast three IDEA models: the original one by Reifman et al. (2007), the theoretical proposal by Arnett’s (2000, 2004), and a reduced proposal put forward by Crocetti et al. (2015). Considering that the different IDEA models have shown limited fit values using confirmatory factor models (Reifman et al., 2016), this study contributes with a more flexible psychometric analysis using the ESEM (Marsh et al., 2014, 2020).

The factorial results in this study showed differences in the fit between the three models. The proposals by Reifman et al. (2007) and Arnett (2000) presented very low fit indices when evaluated by CFA. On the other hand, for these same models, the values in the fit indices improved substantially when evaluated by ESEM, obtaining acceptable values in several indices (see Table 2). In the case of the reduced model proposed by Crocetti et al. (2015), almost acceptable fit indices were observed when evaluated by CFA, and excellent fit indices when evaluated by the ESEM, fulfilling the expectations of Hypothesis 1 and 2.

The IDEA-SV proposal by Crocetti et al. (2015) presented a structure with factorial loadings consistent with the authors’ approaches. However, when evaluated within an ESEM model, small cross-loadings were observed, which were to be expected due to the nature of the Emerging Adulthood construct, embedded in a personality development model (McAdams and Olson, 2010). Thus, the model obtained through ESEM allows corroborating the theoretical structure of the IDEA, presenting high and moderate loadings between the items and their corresponding factors, while taking into account the possible existence of cross-loadings typical of measurements in psychology (Marsh et al., 2014, 2020). Therefore, this model is the one that presents the best fit, for Chilean university students, among the three proposals.

About the correlations between factors, within the ESEM, lower values are generally observed than those of the CFA models (Gomes et al., 2017; Marsh et al., 2020). This is to be expected since CFA models tend to overestimate the correlations between factors, favoring the possible discriminative capacity of each factor by presenting less overlap with correlations between factors without biases. In this way, Hypothesis 3 is confirmed in our data.

Thus, in the present study, the different advantages of this modeling can be demonstrated: it improves the fit (see Table 2); it allows us to check the suitability of the theoretical model to our empirical data to the extent that the main loadings of each factor generally maintain coherence with the theoretical approaches (which occurs in our case, see Table 3); and finally, an additional advantage is that the correlations between the factors of the model are not artificially inflated (see Table 4), as occurs in CFA models (Gomes et al., 2017; Marsh et al., 2014, 2020).

The estimated levels of reliability for the IDEA- SV are considered acceptable, since, although modest values are observed, above 0.60 for each of the five dimensions of the IDEA, it is necessary to take into account that the reliability of a measure is affected by the number of items included in that measure and will depend on the consequences that the score obtained in the test has for the life of the persons. When working in research contexts (American Educational Research Association [AERS], 2014), reliability values considered low can be accepted because the obtained score has little or no effect on the lives of the persons under investigation. Since this situation is presented in this study, Hypothesis 4 is accepted.

Interpretively, a pattern of moderate associations between the IDEA dimensions emerges in this study, as had been reported in the literature (Reifman et al., 2016). Considering that the identity exploration dimension describes the priority task of the stage (McAdams and Olson, 2010), the positive correlations show that the process of identity exploration continues its course as emerging adults perceive themselves ambiguously about their adult status (feeling in between). In this same sense, the incorporation into university spaces, where they meet a wide variety of people in which they visualize evolutionary trajectories different from their own, probably facilitates the experimentation of new behaviors and different ways of life (exploration/possibilities). Consequently, reflection on themselves, their value options, their responsibilities, and the degree of independence developed up to that point (self-focused) is encouraged, all of which are aspects that contribute to the exploration and construction of identity.

Likewise, it is observed that the doubts and hesitations regarding their adult status, which lead them to experiment with the different possibilities presented to them, at the same time translate into instability. This instability shows a low association with identity exploration and, along the same lines, it is observed that as instability increases, experimentation decreases in emerging adults, suggesting a certain degree of paralysis or inhibition, as shown by the weaker correlations, and especially the negative correlation between instability and experimentation.

With these elements, it could be hypothesized that the relationships found in this study correspond to more or less adaptive and healthy patterns of functioning in emerging adults. Thus, there may be relationships between dimensions of the IDEA that reflect healthy and adaptive psychological functioning, such as the relationships between identity exploration, self-focused, and experiencing/possibilities. On the contrary, the relationships between instability/negativity and feeling-in-between, seem to reflect a less adaptive and healthy psychological functioning.

These results provide new evidence of validity for both the theoretical model and the IDEA-SV, verified using an ESEM approach. It is established that the abbreviated version of the IDEA, with 15 items, is the best proposal of the three evaluated; presenting a factorial structure consistent with theoretical expectations, confirming five latent dimensions correlated in a theoretically comprehensible way. This study suggests that these dimensions, besides adequately explaining the correlations between the items of the instrument, contribute to an integrated interpretation that, on the one hand, relates three factors (identity exploration, self-focused, and experimentation/possibilities) that tend to the positive development of EA and two factors (instability/negativity and feeling-in-between) that together point to difficulties or obstacles to wellbeing and development in the course of EA. This proposal, outlined theoretically, must be empirically contrasted in new studies with university emerging adults.

Regarding the limitations, it is important to note that although the study involved a large sample comprising representatives from various public universities across the country, its scope was limited exclusively to university students. Moreover, data collection occurred before the COVID-19 pandemic, an event that, as widely documented, brought significant changes in how society, and particularly university students, interact and cope with the developmental tasks characteristic of this life stage (Ohannessian, 2021; Manze et al., 2021). With this fact in mind, it would be essential to corroborate the findings of this study with a current sample of emergent adults that maintains similar characteristics in terms of size, geographic representation, and age range. Likewise, it would be pertinent to expand the sample to include emerging adults not pursuing university studies, to obtain more generalizable results.

There are future lines of research that may arise from the above. Among them, the validation of characteristics of EA by other associated constructs, which are relevant to study within the higher education context, such as mental health and identity (Crocetti et al., 2008; Crocetti et al., 2015). Also, the impact of cultural aspects on the qualitative and quantitative understanding of emerging adulthood in Chile could be investigated. As suggested by Pérez et al. (2008), it would be relevant to examine the effect of modernization and the predominance of individualistic values on the developmental trajectory of emerging adults. Additionally, future research could explore alternative methods for obtaining scores and developing interpretation standards for the emerging adult population in Chile, by using software such as Factor, Mplus, or the lavaan package in R to estimate individual factor scores. Finally, it is necessary to consider that in comparative studies between groups it will be required to estimate the degree of invariance of the instrument (for example, between university and non-university emerging adults or between different national groups), if it is judged that the instrument documented in this study (IDEA-SV) could be relevant in similar cultural contexts such as those from other Latin American countries.

## Data Availability

The raw data supporting the results and conclusions of this study may be made available upon justified request to the corresponding author.
